# A Mobile App for Triangulating Strategies in Phosphate Education Targeting Patients with Chronic Kidney Disease in Malaysia: Development, Validation, and Patient Acceptance

**DOI:** 10.3390/healthcare10030535

**Published:** 2022-03-14

**Authors:** Lee-Fang Teong, Ban-Hock Khor, Kristo Radion Purba, Abdul Halim Abdul Gafor, Bak-Leong Goh, Boon-Cheak Bee, Rosnawati Yahya, Sunita Bavanandan, Hi-Ming Ng, Sharmela Sahathevan, Sreelakshmi Sankara Narayanan, Zulfitri Azuan Mat Daud, Pramod Khosla, Tilakavati Karupaiah

**Affiliations:** 1 School of Biosciences, Faculty of Health and Medical Sciences, Taylor’s University, Subang Jaya 47500, Malaysia; teongleefang@gmail.com (L.-F.T.); sreelakshmiprem@hotmail.com (S.S.N.); 2 Department of Dietetics and Food Service, Selayang Hospital, Batu Caves 68100, Malaysia; 3 Faculty of Food Science and Nutrition, Universiti Malaysia Sabah, Kota Kinabalu 88400, Malaysia; khorbanhock@gmail.com; 4 School of Computer Science, University of Southampton Malaysia, Iskandar Puteri 79100, Malaysia; kr.purba@soton.ac.uk; 5 Department of Medicine, Faculty of Medicine, Universiti Kebangsaan Malaysia Medical Center, Kuala Lumpur 56000, Malaysia; halimgafor@gmail.com; 6 Clinical Research Center, Serdang Hospital, Kajang 43000, Malaysia; bak.leong@gmail.com; 7 Department of Nephrology, Selayang Hospital, Lebuh Raya Selayang-Kepong, Batu Caves 68100, Malaysia; drbeebc@gmail.com; 8 Department of Nephrology, Kuala Lumpur Hospital, Jalan Pahang, Kuala Lumpur 53000, Malaysia; rosnayahya@gmail.com (R.Y.); sbavanandan@gmail.com (S.B.); 9 School of Medicine, Faculty of Health and Medical Sciences, Taylor’s University, Subang Jaya 47500, Malaysia; nghiming@gmail.com; 10 Department of Dietetics & Nutrition Services, Sunway Medical Center, Petaling Jaya 47500, Malaysia; 11 Department of Allied Health Sciences, Faculty of Science, Universiti Tunku Abdul Rahman, Kampar 31900, Malaysia; sham_0901@yahoo.com; 12 Department of Nutrition and Dietetics, Faculty of Medicine and Health Sciences, University Putra Malaysia, Serdang 43400, Malaysia; zulfitri@upm.edu.my; 13 Department of Nutrition & Food Sciences, College of Liberal Arts & Sciences, Wayne State University, Detroit, MI 48202, USA; aa0987@wayne.edu

**Keywords:** nutrition, mobile app, hemodialysis, hyperphosphatemia, phosphorus, phosphate binder

## Abstract

Hyperphosphatemia afflicts end-stage chronic kidney disease (CKD) patients, contributing to comorbidities and mortality. Management strategies are dialysis, phosphate binder, and limiting dietary phosphate intake, but treatment barriers are poor patient compliance and low health literacy arising from low self-efficacy and lack of educational resources. This study describes developing and validating a phosphate mobile application (PMA). The PMA development based on the seven-stage *Precaution Adoption Process Model* prioritized titrating dietary phosphate intake with phosphate binder dose supported by educational videography. Experts (*n* = 13) first evaluated the PMA for knowledge-based accuracy, mobile heuristics, and clinical value. Adult HD patients validated the improved PMA using the seven-point mHealth App Usability Questionnaire (MAUQ). Patient feedback (*n* = 139) indicated agreement for *ease of use* (69.2%), *interface and satisfaction* (69.0%), and *usefulness* (70.1%), while 72.7% said they would *recommend* this PMA. The *expectation confirmation* for 25 PMA features ranged from 92.1% (*lifestyle*) up to 100.0% (*language option*); and the utilization rate of each feature varied from 21.6% (*goal setting* and *feature-based log*) to 91.4% (*information* on *dietary phosphate* and *phosphate binder*). The Conclusions: *MyKidneyDiet-Phosphate Tracker* PMA was acceptable to adult Malaysian HD patients as part of clinical phosphate management in low-resource settings.

## 1. Introduction

Hyperphosphatemia is prevalent in the end-stage kidney disease population [1]. In Malaysia, approximately 43% of hemodialysis (HD) patients have an above-recommended serum phosphate level >1.8 mmol/L [2]. Hyperphosphatemia is associated with an increased risk of bone disorder, hip fracture, secondary hyperparathyroidism, vascular calcification, cardiovascular disease, and mortality among patients with chronic kidney disease (CKD) [3,4,5,6,7]. The crux of maintaining the patient’s serum phosphate levels within the normal range of 1.1–1.8 mmol/L is via a three-strategy approach inclusive of dialytic phosphate removal, limiting dietary phosphate intake, and using phosphate-binding and other medications [7,8].

HD patients are prescribed high protein diets to offset amino acid losses through the dialysate [9], which will also increase the phosphate load in their diets [10]. Consuming phosphate binders with protein-rich meals is critical to managing serum phosphate levels along with prudent dietary phosphorus restriction [7]. However, these strategies have been shown to be limited in promoting desirable patient behaviors to achieve optimal serum phosphorus control [11]. Issues relate to barriers such as the lack of resources and expertise for patient education [12], poor patient knowledge on phosphate management [13], and phosphate binder medication compliance [11]. It appears that HD patients have the lowest knowledge about dietary phosphorus compared to other nutrients [14,15]. The complexity of phosphate origin compounds is also a determinant of phosphate bioavailability, which is variously affected by organic animal (40–60%) and plant (10–30%) foods with phytates and inorganic (up to 100%) sources [16].

The main three-strategy approach in hyperphosphatemia management requires integrated care from multidisciplinary health carers involving the pharmacist to address phosphate binder use and compliance, the dietitian to deliver the nutrition components of appropriate diets for the patient, and the nephrologist to address the optimization of dialytic procedures, binder dose, and choice [17]. Yet the reality of chronic care delivery for the CKD population globally is a severe shortage of health care personnel, and dietitian access, for which the latter is particularly in shortfall in many countries [18]. In Malaysia, the delivery of nutrition education by dietitians at HD centers nationwide is barely 14.7% for dedicated service and 18% for shared caseloads [19]. Thus, diet counseling primarily depends on physicians and nurses as providers [19,20]. This situation reveals the limitations of effective nutrition education delivery and accuracy of the information in many low-to-middle-income countries [20]. Increasing the number of dietitians could add to the existing healthcare cost burden to dialysis centers [21]. At the same time, nutrition education targeting dietary phosphate control is vital to improve adherence and empower patients to achieve optimal phosphorus and calcium goals [22].

The core issues center on the patient’s lack of knowledge and motivation, lack of accuracy, availability, and accessibility to phosphate information alongside suboptimal phosphate education tools for patients. For instance, didactic phosphate education tools in nutrition are available in Malaysia [23,24,25] but have limited use because they do not inform about phosphate binder choice and dose and physician monitoring. The current practice of fixed phosphate dosing prescription [26] without matching dietary phosphate intake promotes a greater risk of vascular calcification from calcium-based phosphate binder overdose [27]. In this regard, the use of the mobile application (app) to fulfill the nutrition education delivery gaps holds great potential to overcome the conventional mode of education delivery, which is limited in terms of availability, accuracy, and accessibility, as well as lack of renal nutrition specialists.

Nutrition education via a mobile app has produced significantly improved outcomes when engaging with diabetes and weight management [28]. In the field of renal nutrition, several mobile apps have shown improvement in terms of self-monitoring for glucose, inter-dialytic weight gain, and reductions in sodium, potassium, and fluid intakes [29,30,31]. The interest in mobile app use for phosphate management has been gaining momentum with studies from Canada [32,33], United Arab Emirates [34], and Taiwan [35].

An easily accessible and interactive educational tool such as the mobile app to engage in phosphate education to manage and prevent clinical hyperphosphatemia is lacking in Malaysia. Thus, our group aimed to develop a purposive evidence-based mobile app accessible to HD patients and multidisciplinary health care providers incorporating the three-strategy components of patient education necessary in chronic kidney disease-mineral bone disorder (CKD-MBD) management. We recognized that to be a useful educational tool, the mobile app should provide accurate and updated information on phosphate management for HD patients in aural and visual infographics [36]. Simultaneously, the tool should primarily consist of local food choices enabling diet planning and titrating with phosphate binder prescription [37]. The tool should also include the feature to support self-monitoring and self-efficacy [38]. Finally, the app should be validated and accepted by both caregivers and the patient population. This paper reports on the development, validation, and acceptance of a mHealth mobile app termed in this paper as the Phosphate Mobile App (PMA) for Malaysian HD patients, as an innovative approach to narrowing the phosphate education gaps outlined above.

## 2. Materials and Methods

The theoretical principle underpinning this PMA development was based on the seven-stage *Precaution Adoption Process Model*, which applies the stages of change in health behaviors to phosphate management in HD patients [39]. The model discerns modifiable and non-modifiable factors influencing individual health beliefs system (*thought*), affecting dietary and lifestyle patterns (*behavior*) and emotion, resulting in serum phosphorus levels as an outcome [39].

### 2.1. Development of Phosphate Mobile App (PMA)

**Figure 1:**
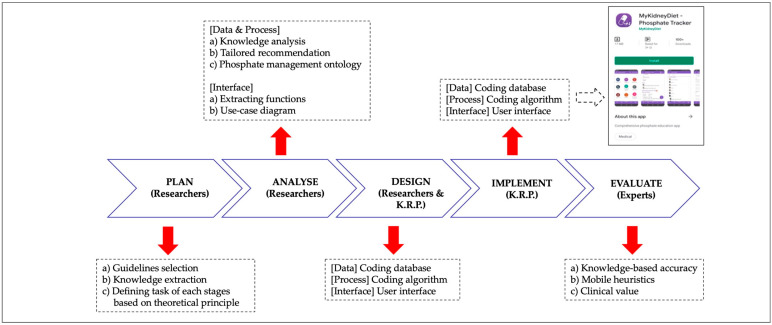
Development of the Phosphate Mobile App. Researchers: T.K., L.F.T., B.H.K., Z.A.M.D., H.M.N., S.S., A.H.A.G., R.Y., B.L.G., S.B., and B.C.B.

The development of the PMA was initiated by six dietitians (T.K., L.F.T., B.H.K., Z.A.M.D., H.M.N., and S.S.) and five nephrologists (A.H.A.G., R.Y., B.L.G., S.B., and B.C.B.). The study followed institutional ethical guidelines defined by the Taylor’s University Human Ethics Committee (HEC 2019/011). The development involved planning, analysis, design, implementation, and evaluation adapted from a Korean web-Roadmap model [40]. Each development step included data, process, and interface designation. The overall flow in the design and development is conceptualized in Figure 1.

#### 2.1.1. Planning of the PMA

Eleven strategies from health belief theories were determined in accordance with the *Precaution Adoption Process Model*, and these were consolidated into six (6) key domains relevant to phosphate education as listed in Table S1. Knowledge in the context of serum phosphorus management was extracted from guidelines with additional expert opinion included, and strategies were then determined for each component and domain of interest according to expert recommendations. The guidelines that served to benchmark this PMA’s development were KDIGO Clinical Practice Guideline Update for the Diagnosis, Evaluation, Prevention and Treatment [7], Malaysian CKD-MBD and Parathyroidectomy Guidelines and Standard Operating Procedures [8], CKD 5D nutrition management–Chronic Kidney Disease Guideline 2010 [41], and Dialysis patients’ responsibilities–Bill of Rights and Responsibilities [42]. Strategic domains with criteria benchmarked to the guidelines and expert opinion are outlined in Table S2.

Two interactive components of the PMA, as determined by the strategies and criteria for phosphate management, required the assessment of dietary intake and phosphate binder use. The required databases are described below:

**Food database**—This was built from the ongoing Palm Tocotrienol in Chronic Hemodialysis Study, in which 388 adult HD patients’ dietary intake records participating in the screening study were utilized. The 3-day dietary intakes comprised 24-h dietary recalls for one weekend day, a non-dialysis weekday, and a dialysis weekday. The nutrient values used in this food database were derived from the Malaysian Food Composition^4^ and Singapore Food Composition^5^ databases. The PMA food choices reflected typical food intake patterns of Malay, Chinese, and Indian patients living in the Klang Valley [43,44].

**Phosphate binder calculation algorithm**—The PMA algorithm was built based on the following assumptions:

- Intestinal absorption for inorganic phosphate sources is 100%, 50% for animal-based dietary phosphate (40–60%), and 10–30% for plant-based dietary phosphate [16]. For a mixed meal (containing more than one dietary phosphate source), the higher intestinal absorption value as per individual food components was referenced.
- Dialysis treatment removes about 2300–2600 mg phosphate per week with the standard 12 h HD treatment a week and blood flow achieving a dialysis blood quotient (Qb) of 300 mL/min on average [45].
- Phosphate binding capacity for calcium carbonate is 19.0 mg per 500 mg tablet and for lanthanum carbonate is 67.5 mg per 500 mg tablet and for sevelamer is 21.4 mg per 800 mg tablet [46].
- Patients have about 40% phosphate removal through bowel output (feces) and do not have constipation issues [47].

#### 2.1.2. Analytical Procedures of the PMA

A use-case diagram was derived for users’ interactions with the PMA system, with the involvement of the various users (Figure S1).

#### 2.1.3. Design of the PMA

Incorporating strategies into the PMA required specific features, such as:

- Education videography regarding phosphate and dialysis lifestyle;
- Interactive self-efficacy features included food and beverages nutrient content and choices to reduce dietary phosphate load, goal setting in blood profiles and weight, self-monitoring of dietary phosphate intake, dialysis treatment, phosphate binder use, emotion, exercise and weight, and phosphate calculation for serum phosphorus reduction; and
- Interactive cues to action features included phosphate binder intake reminders and dietary phosphate and phosphate binder adjustments.

An entity-relationship diagram was created to build a data domain from the data extracted in the planning and analysis phase (Figure S2).

#### 2.1.4. Implementation of the PMA

The development of the PMA by a professional information technologist (K.R.P.) was materialized with the database, algorithm, and user interface using coding and an independent online server with security encryption. The PMA prototype was supported in three languages (English, Malay, and Mandarin). The PMA interface was organized into five main sections as follows:

- *Profile*—providing the user profile information.
- *Information*—providing seven features of education, which include graphical household *Measurement* tools, and videography on *Phosphate, Dialysis, Phosphate Binder, Dietary Phosphate, Lifestyle,* and *Responsibility*.
- *Input*—providing nine features that supported self-efficacy and cues to action, for *Food/drink*, *Treatment, Blood test, Weight*, *Phosphate binder,* Phosphate binder *reminder*, *Exercise, Emotion,* and *Phosphate calculator*.
- *Log*—providing three features for record-keeping that come in different formats of *Daily, Periodic,* and *Feature-based* log.
- *Setting*—providing six features facilitating users for *Unit converter, Goal setting*, *FAQ, Feedback*, *Adjust font*, and *Language option.*

#### 2.1.5. Validation of the PMA by Expert Reviewers

A beta version of the PMA was validated by the expert reviewers on the knowledge-based accuracy, mobile heuristics, and clinical value. One-on-one semi-structured qualitative interviews in English were carried out with reviewers during a 1-month validation (September 2019) period. Inclusion criteria for reviewer selection targeted nephrologists, senior pharmacists, or senior dietitians with a minimum of five years of clinical experience or research in their specialized fields. Essentially, reviewers were selected through purposive sampling [48], ideally located in the Klang Valley and accessible to the researcher (L.F.T.) during the 1-month validation period. Reviewers had to provide constructive feedback for the PMA within this period.

The knowledge-based accuracy in the PMA was benchmarked to recommendations from guidelines and previous studies, the standard of practice, and in terms of the practicality of recommendations to the Malaysian clinical setting [7,8,41,42]. The content evaluation covered the food database, education videos, and information provided in the PMA. Inconsistencies and misinformation that emerged in the evaluation feedback were used to improve the PMA system. Revised statements were grouped into corrected, improved, or added categories according to the six key domains, namely phosphate and hyperphosphatemia, dialysis, phosphate binder, dietary phosphate, lifestyle, and responsibility as a dialysis patient.

For mobile heuristics evaluation, the reviewers were asked to comment on usability issues related to eight heuristics components given the interactive design of the PMA. Each heuristic issue was ranked based on Nielsen’s severity ranking scale from one (0) to four (4), with zero (0) as ‘no usability problem’ and four (4) as ‘usability catastrophes’, which are imperative to rectify before the product could be released. The heuristic issues were indicated as faulty if two or more people scored them as one (1) point or higher on the severity ranking scale. The PMA system was revised for these items according to the ability of the app developer [49].

For subjective clinical value evaluation of the PMA, two close-ended questions, ‘Do you think this app will be helpful for dialysis patients in phosphate management?’ and ‘Do you think this app will be helpful for health care providers in phosphate management?’, with ‘yes’ and ‘no’ answers, were asked of the reviewers.

All feedback from experts was ratified for PMA improvement to finalize the PMA prototype, which was to be validated by adult HD patients.

### 2.2. Evaluation of PMA Acceptance by Adult HD Patients

#### 2.2.1. Study Design

A cross-sectional study was conducted with adult HD patients to evaluate acceptance and perception of a prototype version of the PMA formally registered as *MyKidneyDiet–Phosphate Tracker*. The evaluation was conducted between January 2020 to March 2020. All patients provided written informed consent to participate in the study, which was approved by the Medical Research Ethics Committee of the Ministry of Health, Malaysia (NMRR-19-3825-51342).

#### 2.2.2. Patient Recruitment

All adult HD patients attending 10 participating HD centers in the Klang Valley were screened for eligibility using universal sampling. Inclusion criteria for eligibility were patients aged ≥18 years old, on maintenance HD, and able to read English, Malay, or Mandarin. Essentially all patients had to own and/or be able to use a smartphone, were prepared to download and use the PMA independently, and had access to the internet. Patients with cognitive or visual disabilities were excluded. Consenting eligible patients were recruited until the expected sample size was achieved. Recruited patients were assisted with the PMA installation on their smartphones and had to provide feedback on the PMA use after two weeks of trial via face-to-face interview sessions with the researcher.

#### 2.2.3. Demographics, Clinical, and Mobile App Experience

Patients reported their age, ethnicity, marital status, education level, monthly income, and employment. Information on dialysis initiation date for dialysis vintage (month), dialysis center sector, and type of phosphate binder were obtained from patient medical records. The type of mobile app used, its smartphone operating system, smartphone usage during HD treatment (minutes), and previous exposure to other nutrition app use were also ascertained.

#### 2.2.4. Outcome Measures

**Acceptance and Perception of the PMA**—The mHealth App Usability Questionnaire (MAUQ) was used to determine patients’ acceptance and perception towards the PMA as the primary outcome [50]. The MAUQ was adapted from a validated study for a standalone healthcare-related mobile app appropriate to skincare for both patients and health care provider settings. The questionnaire included 18 items from MAUQ, derived from 3 domains relating to ease of use (5 items), interface and satisfaction (7 items), and usefulness (6 items) [50]. An additional item ‘I would recommend this app to my friend on dialysis’ was added by us. The patients would rate each item using a seven-point Likert scale with one (1) as strongly agree and seven (7) as strongly disagree. The original author [50] granted permission to use the MAUQ in this study.

**Expectation Confirmation of the PMA Features**—Expectation confirmation projects the users’ tendency or intention to continue using the PMA feature [51]. A feature-based expectation confirmation checklist was, therefore, used to rate the 25 PMA features as the secondary outcome. The 25 PMA features determined are 7 features of *Information,* 9 features of *Input,* 3 features of *Log,* and 6 features of *Setting.* The study adopted the modified statement of *‘My experience using the following features in MyKidneyDiet-Phosphate Tracker was better than what I expected*’ [52]. The patients rated each item using a seven-point Likert scale with one (1) as strongly agree and seven (7) as strongly disagree. Patients were advised to rate only if they had tried the features.

**Self-reported Utilization of PMA Features**—Feedback was extracted from the expectation confirmation checklist for 25 features in the PMA, with each feedback considered as a utilization.

#### 2.2.5. Statistical Analysis

Data from the study were collected and stored in an encrypted computer for data analysis purposes, and access was allowed only to researchers (T.K. and L.F.T.) involved in the data analysis. Incomplete data were not analyzed.

Descriptive continuous variables were presented as mean ± standard deviation for age with normal distribution and median (interquartile, IQR) for dialysis vintage (month). Categorical variables were expressed as frequency (percentages). The rating from the MAUQ, recommendation, and PMA feature expectation confirmation were reported in percentage, with ratings of ‘1’ to ‘3’ considered as agreement, ‘4’ as neutral, and ‘5’ to ‘7’ as disagreement. The self-reported 25-PMA feature utilization pattern extracted from the expectation confirmation data was described in percentage (%).

The statistical analysis of this study was performed using the IBM SPSS Version 26 (IBM SPSS Statistics Inc., Chicago, IL, USA, 2019).

## 3. Results

### 3.1. Phosphate Mobile App

The *MyKidneyDiet-Phosphate Tracker* PMA installation required 25 megabytes of memory space and was compatible with Android 4.4 and above applications for the Google Play Store, as well as required 49 megabytes of memory space iOS 12.0 or later versions for the Apple App Store. The PMA features enabled offline mode use, whereas data synchronization and educational videos required internet access. Initially, the food database was restricted to food patterns yielding 335 foods prevalent amongst the Klang Valley HD population [43,44]. However, the continuous virtual real-time updates of foods not in the database resulted in 471 new food items added through the users’ feedback.

**Figure 2:**
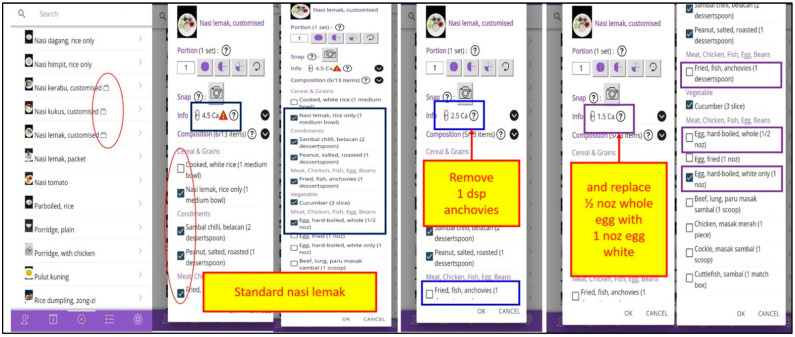
Interactive features of the MyKidneyDiet–Phosphate Tracker targeting lower dietary phosphate load food item selection. Abbreviation: dsp, dessertspoon.

Figure 2 indicates the information screen on the *MyKidneyDiet-Phosphate Tracker* app projecting the interactive feature for titrating the required phosphate binder dose with the dietary phosphate content of the selected food/meal. The feature allows users to replace food items with high dietary phosphate load with choices carrying low dietary phosphate load, to enable lowering the overall dietary phosphate content in that meal to accommodate the prescribed phosphate binder.

### 3.2. Experts Feedback

The PMA feature was validated by thirteen experts who were nephrologists (*n* = 5), renal pharmacists (*n* = 2), and senior dietitians (*n* = 6) from both public (*n* = 11) and private (*n* = 2) institutions. However, physicians (*n* = 5) affiliated with the government were also affiliated with non-government or private HD sites. Most panel members had been practicing for 11 to 19 years in their respective fields. The evaluation experts were representative of different age groups and major Malaysian ethnicities, namely Malay, Chinese, and Indian. Women members were double their male counterparts in the panel. Their demographic characteristics are presented in Table S3. Their assessment was as follows:

**Knowledge-Based Accuracy**—In total, 43 feedback comments required revision, which was mainly concerned statement improvement (*n* = 20), statement enhancement (n = 16), and statement correction (*n* = 7). All data are presented in Table S4.

**Mobile Heuristics**—A total of 46 issues relating to the PMA were raised by the expert panel as *major usability problems* (*n* = 17), *minor usability problems* (*n* = 17), and *cosmetic problems* (*n* = 12). No issue was raised for the severity ranking scale rating scores of zero (*not a usability problem*) and four (*usability catastrophes*). The most frequent issues highlighted were for the *ease of input, screen readability, and glanceability heuristic* (*n* = 14), followed by the *match between system and the real-world heuristic* (*n* = 8) and the *aesthetic, privacy, and social conventions heuristic* (*n* = 7). All data are presented in Table S5. All heuristics issues raised by the reviewers were discussed with the PMA developer (R.P.K.) and were revised accordingly before the validation of the PMA prototype by patients.

**Clinical Value**—All experts unanimously agreed that this PMA would be helpful for both dialysis patients and health care providers in phosphate management.

### 3.3. Evaluation of PMA Acceptance by HD Patients

A total of 369 adult HD patients from six HD centers in the Klang Valley were assessed for eligibility using universal sampling. However, only 172 patients were recruited for the PMA validation. The main reasons for exclusion (51.2%, *n* = 189) were patients were unable to handle interactive mobile applications independently (*n* = 122), outdated smartphone operating system (*n* = 38), had poor eye vision (*n* = 18), and/or had smartphones that lacked memory space (*n* = 11). After recruitment, patient dropouts (19.2%, *n* = 33) also occurred due to the COVID-19 pandemic in Malaysia (starting March 2020) if they were dialyzing at HD centers located in the red zone areas of the Klang Valley, which were under lockdown. Data analyses, therefore, were based on only 139 patients who completed the PMA testing and the survey.

**Patient background**—The mean age of patients was 48.1 ± 13.2 years, with a dialysis vintage median (IQR) of 72 (76) months (Table 1). The patient population was equally representative for gender distribution (males = 50.4%; females = 49.6%). As per ethnic distribution, 46.8% were Malay, 36.0% were Chinese, 15.8% were Indian, and 1.4% were other ethnicities.

HD patients involved in this study were mainly dialyzing at a non-government (NGO) site (48.9%), followed by government (30.2%) and private (20.9%) centers. A significant proportion of the HD patients was on calcium carbonate (89.2%), followed by non-calcium-based phosphate binders, which are sevelamer carbonate (5.8%), and lanthanum carbonate (3.6%), while 1.4% were not on any phosphate binder. Out of 139 adult HD patients, 93.5% were using smartphones supported by the *Android* operating system, and 6.5% were on the *iPhone* operating system. During their weekly three-day HD treatment, 89.2% of patients would use smartphones during each 4-h dialysis session. The median (IQR) time on using smartphones by patients was approximately 120 (105) minutes per dialysis session, equivalent to 6 h per week.

**Acceptance and Perception of *the PMA by* Patients**—Based on patients’ evaluation, median (IQR) values were 2.6 (2.0) for *ease of use*, 2.6 (1.9) for *interface and satisfaction*, 2.5 (2.0) for *usefulness* for the MAUQ, and 2.0 (3.0) for *recommendation* (Table 2). The ratings were toward agreements for each evaluation. Further exploring the domain of *ease of use,* 69.8% of surveyed patients deemed the PMA was *easy to use*, 71.2% found the PMA was *easy to learn to use*, 70.5% indicated that the *PMA navigation was consistent*, 67.6% claimed that the *PMA interface allowed them to use all the functions*, and 66.9% stated *they could recover quickly from their mistake in the app use*. For the domain of *interface and satisfaction*, around 69.1% *liked the PMA interface*, 69.1% noted *information in this PMA was well-organized*, 70.5% acknowledged that the *PMA allowed them to know their progress*, 70.5% were *comfortable to use the PMA in social settings*, 66.2% felt the *amount of time spent using this PMA was fitting*, 74.8% patients would *want to use the PMA again*, and 71.2% adult HD patients were *satisfied with this PMA*. For the domain of *usefulness*, about 74.1% of adult HD patients recognized this PMA as *useful for their healthcare practice*, 72.7% believed this PMA would *improve their access to healthcare*, 69.8% considered this PMA would *help them managed their health effectively*, 69.8% agreed that this PMA *had all the functions that they expected it to have*, and 56.1% could *use the PMA even when the internet connection was poor or unavailable*. About 72.7% of the patients considered this PMA as *an acceptable way to receive health care services* and would *recommend this PMA to their dialysis colleagues*.

**Expectation Confirmation of the PMA Features**—Patients rated several features with a median (IQR) of 1.0 (1.0), which were informative videos (*Phosphate, Dialysis, Phosphate Binder, Dietary Phosphate, Lifestyle, Responsibility*), *Input tools* (food/drink, treatment, blood test, phosphate binder, reminder, emotion and exercise, phosphate calculator), *logs* (daily and feature-based), and all *Setting* features (*Language Option, Unit Converter, Goal-Setting, FAQ, Feedback,* and *Font Adjustment*). *Weight, Measurement,* and *Periodic log* features were rated 2.0 (1.0) for the expectation-confirmation rating (Table 3). The ratings were towards agreements for the feature expectation.

**Figure 3:**
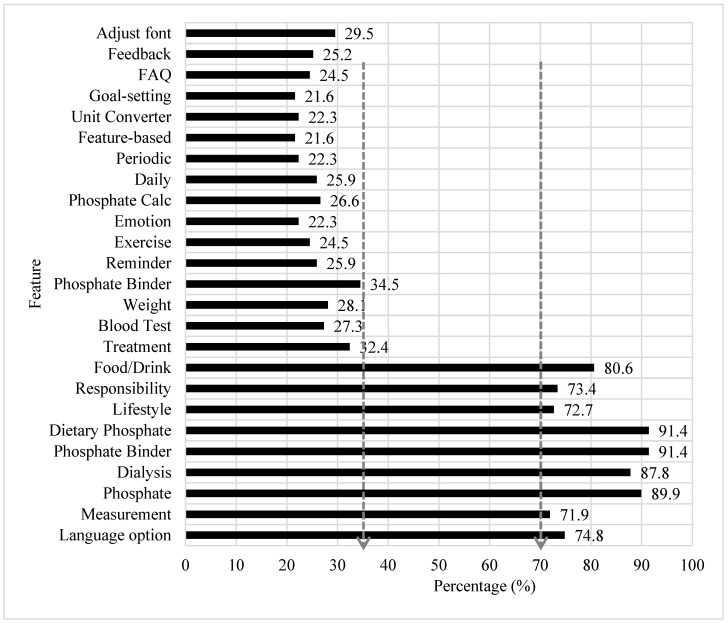
Self-reported rating for the 25-PMA feature utilization.

**Self-reported PMA Feature Utilization**—For the 25 features of the PMA, utilization ranged from the highest 91.4% for videography on *dietary phosphate* and *phosphate binder,* to the lowest 21.6% for *goal-setting* and *feature-based log*. Two distinctive utilization patterns were discretely observed as above 70% utilization and less than 35% utilization (Figure 3).

## 4. Discussion

Our study detailed the development of the *MyKidneyDiet-Phosphate Tracker* PMA, which experts then validated to improve the PMA before we proceeded to the final evaluation step for acceptance by adult HD patients. This PMA is a novel mobile app developed for the HD population in Malaysia, with designated features in concordance with the theoretical principles underpinning the *Health Belief Model* integrated into the seven-stage process steps of the *Precaution Adoption Process Model* [39]. The integration of modifiable and non-modifiable factors influencing patients’ knowledge and practices regarding managing serum phosphorus levels motivated the PMA development with the partisanship of relevant healthcare providers and patients. The ultimate objective of the PMA is to promote health literacy, which is critical to patient empowerment through access to health information and patient capacity to use it effectively [53].

Accurate and precise diet information is critical to effective dietary self-management in CKD patients. In present times, patients are vulnerable to lack of information accuracy on the renal diet, which could be 73.0% if originating from websites or even as low as 18.0% from YouTube videos [54]. Thus, the initial validation of the PMA for content accuracy, mobile heuristics, and clinical value by the 13 multidisciplinary experts representing nephrologists, dietitians, and pharmacists was an essential step in the PMA development, as the clinical relevance and healthcare stakeholders’ usability of the app is very critical to the objective of facilitating hyperphosphatemia management. These stakeholder feedbacks necessitated further improvement of the PMA. In contrast, two studies reporting on mobile apps development sharing the same objective as our study did not detail expert validation [34,55].

After completion of the expert validation, the *MyKidneyDiet-Phosphate Tracker* prototype was facilitated through the Google Play Store and Apple App Store. This version underwent the patient validation protocol. As assessed by the MAUQ, the HD patients validating the PMA were in favor of the app designated by the agreement towards acceptance as per *ease of use* (69.2%), *interface and satisfaction* (69.0%), *usefulness* (70.1%), and *recommendation* (72.7%). Our engagement with the MAUQ to measure acceptance is for the first time been reported in the literature. Two other studies on PMAs specific to the HD patient population were the *KELA.AE* in the Arabic language relevant to the United Arab Emirates [34] and *Easy Diet Diary Renal^TM^* in the English language in Australia [56]. The *KELA.AE* PMA was validated with only 23 adult HD patients and focused on PMA efficacy instead of acceptance and usability. In contrast, the *Easy Diet Diary Renal^TM^* PMA focusing on sodium and phosphate was validated with 25 healthy adults, who assessed acceptance informally without structured questions. Since very few studies assess the acceptance of PMAs in CKD populations, an analogy may be drawn with other digital tools delivering education. Usability and acceptance of mHealth digital tools evaluation in the field of kidney disease by CKD patients were mostly reported for personal digital assistants (PDAs) [57,58,59], mobile phone text [60], and mobile electronics [61,62]. These studies used (i) the 25-item Rawl usability questionnaire with a modified five-point Likert scale [61,62], (ii) an intervention tool compliance assessment in terms of usage frequency [58,59], or iii) a single-item usefulness scale from 0 (not useful at all) to 10 (extremely useful) [60]. Most of these studies concurred that the investigated tools were helpful, useful, or acceptable by their respective target groups.

As regards the expectation confirmation of the PMA features, *information* achieved agreement from 92.1 to 96.0%, *input* attained 93.7% to 97.9%, *log* obtained 96.7% to 97.2% and *setting* had 94.3% to 100.0%. With all features having expectation confirmation above 90.0%, along with the *language option* feature having 100.0% expectation confirmation, each feature in this PMA fulfilled the expectation for its purpose. However, this study outcome might not reflect actual opinion as patients are more likely to express a positive attitude towards mHealth features, as observed in a mHealth literacy survey carried out among 4504 public respondents in Selangor, Malaysia [63]. Moreover, we noted hardly any research published on the expectation confirmation assessment for individual features of the PMA or even mHealth digital tools for general health or even disease-specific patient education.

We further explored patient perceptions as regards the utilization pattern of the *MyKidneyDiet-Phosphate Tracker* features. There was a distinctive self-reported PMA feature utilization pattern emerging with one group of utilization patterns above 70% utilization and the other less than 35% utilization. The features with above 70% utilization were *language option* (74.8%), *information* (71.9% to 91.4%), and *food/drink* (80.6%). The higher utilization pattern of the PMA was attributed to its availability in three languages options (English, Malay, and Chinese) on the *Google Play Store* and *Apple App Store*, which is reported for the first time worldwide for an mHealth and kidney-related mobile app. Another reason for higher utilization by patients was the education in the knowledge domain that was delivered pictorially with household measurements of food portions and adopting a video format with audio and subtitles for specific topics instead of conventional text narratives. Importantly, high utilization was also tied to titration features for phosphate binder requirement and adjustment for a meal, and a customized menu composition selection to adjust for dietary phosphate reduction.

In contrast, PMA features with less than 35% utilization were associated with log features, as traditionally, Malaysian patients have low health literacy towards self-efficacy [53]. Moreover, only seven patients (5%) in this studied population had experience with previous nutrition app use, as reported in Table 1. Another reason for the low pattern of utilization by the patients was related to the complexity of function specific to the *Phosphate calculator* (*n* = 37). This feature was targeted for healthcare providers and HD patients with higher literacy and understanding of disease management, and related to advanced phosphate binder dosing and dietary phosphate reduction management. Another plausible reason for the low utilization rate by the patients may be attributed to the short duration of two weeks given for the PMA trial, which meant insufficient time for trialing features such as the *periodic log*. Of note, the nine *Input* tools of the PMA were accounted by the theoretical model adopted for the PMA development aiming to encourage and assist patients’ *self-efficacy* and *cues to action* [64]. Nevertheless, low levels of ‘self-efficacy’, ‘lack of readiness to change’, and/or ‘awareness of the issue’ according to the Health Belief Model [65], may explain why our HD patients were not ready or not keen to use these features. Therefore, additional strategies for other modifying factors are critical for patients’ empowerment to promote self-efficacy [39].

There are some limitations to this study. Firstly, the patient validation of the PMA was conducted for the Klang Valley, which only represented an urbanized HD population, and did not include HD patients from rural areas. However, results from this validation may be extrapolated to the overall Malaysian HD population, as more than 70% of Malaysians are dialyzing in the urban areas judging from 667 HD centers registered with the Malaysia National Renal Registry in 2015. Secondly, the patient validation involved patients from a younger age group (45 to 54 years), whereas the majority of the adult Malaysian HD patients were older (55 to 64 years) [2]. However, one eligibility criteria for participation was smartphone usage, which excluded the older age group. Therefore, the HD population not ready for PMA use will still have to depend on conventional dietetic counseling supported by phosphate patient information sheets.

Overall, we have detailed concepts in the development of this PMA, which will serve to guide other researchers interested in mobile health development for disease management. Of note, the nutrition education videos embedded in this PMA are necessary to mitigate the lack of dietitian access in Malaysia. A notable feature was the PMA facilitated the needs of Malaysia’s multi-ethnic population in terms of language and food diversity, which explained the PMA’s high acceptance rate with patients. We perceive the usefulness of the PMA in clinical settings, where its interactive features are critical for promoting self-efficacy in CKD patients as well as enabling phosphate binder dosing by clinicians. This PMA should potentially be implemented as a comprehensive educational tool for CKD patients with hyperphosphatemia due to its convenience of use. From this study’s experience, we found that training was crucial for the HD patients in Malaysia and HD health care providers to increase the utilization rate and awareness of the PMA. Above all, the PMA development embodies the need of the integrated care model in health care management to benefit patient centered care.

## 5. Conclusions

Overall, our acceptance evaluation revealed the *MyKidneyDiet-Phosphate Tracker* PMA is acceptable to patients and should be optimized as a phosphate education tool in HD centers without access to renal dietitian services, or to complement these services. However, this PMA will be limited to patients who have access to smartphone devices and can use smartphones independently or to be assisted in this. Future studies are warranted to confirm the efficacy of this PMA in managing hyperphosphatemia in HD patients.

## Figures and Tables

**Table 1:** Patient socio-demographics (*n* = 139).

Parameter	Mean ± SD	*n* (%)
Age	48.1 ± 13.2	
^ꝉ^ HD vintage (month)	72 (76)	
Gender		
Male		70 (50.4)
Female		69 (49.6)
Ethnicity		
Malay		65 (46.8)
Chinese		50 (36.0)
Indian		22 (15.8)
Others		2 (1.4)
Marital status		
Married		105 (75.5)
Single		29 (20.9)
Divorced		5 (3.6)
Education level		
Diploma/Degree/Higher		57 (41.0)
Secondary		72 (51.8)
Primary		10 (7.2)
Monthly income		
<RM1000		68 (48.9)
RM1001–RM3000		47 (33.8)
RM3001–RM5000		13 (9.4)
>RM5000		11 (7.9)
Employment		
Working		50 (36.0)
Not working		89 (64.0)
Sector (Dialysis center)		
Government		42 (30.2)
Non-government		68 (48.9)
Private		29 (20.9)
Type of phosphate binder		
Calcium carbonate		124 (89.2)
Sevelamer carbonate		8 (5.8)
Lanthanum carbonate		5 (3.6)
Not on phosphate binder		2 (1.4)
Operating System		
Android		130 (93.5)
iPhone		9 (6.5)
Smartphone use during HD treatment		
Yes		124 (89.2)
No		15 (10.8)
^ꝉ^ If yes, mobile app use during HD (minute/session)	120 (105)	
Type of mobile app use during HD *		
Watch video		85 (61.2)
Social interaction		78 (56.1)
Games		38 (27.3)
Listen to music		36 (25.9)
Educational & information seeking		25 (18.0)
Previous nutrition apps use		
Yes		7 (5.0)
No		132 (95.0)
Challenges of nutrition app use *		
Features do not meet my expectation		3 (2.2)
Information is too general		3 (2.2)
No new information		1 (0.7)
Reason for not using a nutrition app *		
I am not aware of the app in the app store		102 (77.3)
Features offered do not meet my expectation		24 (18.2)
Not interested in a nutrition app		4 (3.0)
I have to pay for it		1 (0.8)
Not enough phone storage		1 (0.8)

Values are expressed as mean ± 1 SD and frequency (percentage), except where indicated. ^ꝉ^ Data are presented as median (interquartile range). * Patients could choose more than one option.

**Table 2:** Acceptance response based on the mHealth App Usability Questionnaire (MAUQ) and recommendation (*n* = 139).

No.	Statement	*n* (%)		
		Agree	Neutral	Disagree
**Ease of use** ^a^		**96 (69.2)**	**26 (18.7)**	**17 (12.1)**
1	The app was easy to use.	97 (69.8)	27 (19.4)	15 (10.8)
2	It was easy for me to learn to use the app.	99 (71.2)	24 (17.3)	16 (11.5)
3	The navigation was consistent when moving between screens.	98 (70.5)	23 (16.5)	18 (13.0)
4	The interface of the app allowed me to use all the functions (such as entering information. Responding to reminders, viewing information) offered by the app.	94 (67.6)	29 (20.9)	16 (11.5)
5	Whenever I made a mistake using the app, I could recover easily and quickly.	93 (66.9)	27 (19.4)	19 (13.7)
**Interface and satisfaction** ^a^		**96 (69.0)**	**30 (21.7)**	**13 (9.3)**
6	I like the interface of the app.	96 (69.0)	29 (21.0)	14 (10.0)
7	The information in the app was well organized, so I could easily find the information I needed.	96 (69.0)	31 (22.3)	12 (8.7)
8	The app adequately acknowledged and provided information to let me know the progress of my action.	98 (70.5)	28 (20.1)	13 (9.4)
9	I feel comfortable using this app in social settings.	98 (70.5)	33 (23.7)	8 (5.8)
10	The amount of time involved in using this app has been fitting for me.	92 (66.2)	30 (21.6)	17 (12.2)
**Usefulness** ^a^		**98 (70.1)**	**25 (18.1)**	**16 (11.8)**
11	I would use this app again.	104 (74.8)	24 (17.3)	11 (7.9)
12	Overall, I am satisfied with this app.	99 (71.2)	28 (20.1)	12 (8.7)
13	The app would be useful for my health care practice.	103 (74.1)	21 (15.1)	15 (10.8)
14	The app improved my access to health care services.	101 (72.7)	21 (15.1)	17 (12.2)
15	The app helped me manage my health effectively.	97 (69.8)	23 (16.5)	19 (13.7)
16	This app has all the functions and capabilities I expected it to have.	97 (69.8)	21 (15.1)	21 (15.1)
17	I could use the app when the Internet connection was poor or not available.	78 (56.1)	38 (27.3)	23 (16.6)
18	This app provided an acceptable way to receive health care services, such as accessing educational materials, tracking my own activities, and performing self-assessment.	101 (72.7)	25 (18.0)	13 (9.3)
Recommendation *				
19	I would recommend this app to my friend on dialysis.	101 (72.7)	27 (19.4)	11 (7.9)

^a^ Values in bold are overall scores of the domains. * Additional item added to MAUQ (18-item).

**Table 3:** Expectation confirmation of PMA features.

Feature	Utilize ^a^	Likert Scale ^a^		
		Agree	Neutral	Disagree
Information				
Measurement	100 (71.9)	96 (96.0)	4 (4.0)	0 (0.0)
Phosphate	125 (89.9)	119 (95.2)	3 (2.4)	3 (2.4)
Dialysis	122 (87.8)	115 (94.2)	3 (2.5)	4 (3.3)
Phosphate binder	127 (91.4)	120 (94.5)	4 (3.1)	3 (2.4)
Dietary phosphate	127 (91.4)	120 (94.5)	4 (3.1)	3 (2.4)
Lifestyle	101 (72.7)	93 (92.1)	6 (5.9)	2 (2.0)
Responsibility	102 (73.4)	96 (94.1)	2 (2.0)	4 (3.9)
Input				
Food/drink	112 (80.6)	105 (93.7)	4 (3.6)	3 (2.7)
Treatment	45 (32.4)	43 (95.6)	1 (2.2)	1 (2.2)
Blood test	38 (27.3)	37 (97.4)	1 (2.6)	0 (0.0)
Weight	39 (28.1)	37 (94.9)	2 (5.1)	0 (0.0)
Phosphate binder	48 (34.5)	47 (97.9)	1 (2.1)	0 (0.0)
Reminder	36 (25.9)	34 (94.4)	2 (5.6)	0 (0.0)
Exercise	34 (24.5)	33 (97.1)	1 (2.9)	0 (0.0)
Emotion	31 (22.3)	30 (96.8)	1 (3.2)	0 (0.0)
Phosphate calculator	37 (26.6)	36 (97.3)	1 (2.7)	0 (0.0)
Log				
Daily	36 (25.9)	35 (97.2)	1 (2.8)	0 (0.0)
Periodic	31 (22.3)	30 (96.8)	1 (3.2)	0 (0.0)
Feature-based	30 (21.6)	29 (96.7)	1 (3.3)	0 (0.0)
Setting				
Unit converter	31 (22.3)	30 (96.8)	1 (3.2)	0 (0.0)
Goal setting	30 (21.6)	29 (96.7)	1 (3.3)	0 (0.0)
FAQ	34 (24.5)	33 (97.1)	1 (2.9)	0 (0.0)
Feedback	35 (25.2)	33 (94.3)	2 (5.7)	0 (0.0)
Adjust font	41 (29.5)	40 (97.6)	1 (2.4)	0 (0.0)
Language option	104 (74.8)	104 (100.0)	0 (0.0)	0 (0.0)

^a^ Values are expressed as *n* (%). Abbreviation: FAQ = Frequently Asked Question; PMA—Phosphate Mobile Application.

## Data Availability

The datasets generated and analyzed for the current study are available from the corresponding author, T.K., upon reasonable request.

## Supplementary Materials

The following supporting information can be downloaded at: https://www.mdpi.com/article/10.3390/healthcare10030535/s1, Figure S1: Use-case diagram of the PMA; Figure S2: Entity-relation diagram of the PMA. Table S1: Strategies of hyperphosphatemia management for PMA development; Table S2: Criteria of management domains for hyperphosphatemia; Table S3: Characteristics of Expert Reviewers; Table S4: Knowledge-based accuracy feedback from the expert panel; Table S5: Knowledge-based accuracy feedback from the expert panel.

## References

[1] Vervloet M.G., van Ballegooijen A.J. (2018). Prevention and treatment of hyperphosphatemia in chronic kidney disease. Kidney Int..

[2] Wong H.S., Goh B.L. (2018). Twenty Forth Report of the Malaysian Dialysis and Transplant 2016.

[3] Palmer S.C., Hayen A., Macaskill P., Pellegrini F., Craig J.C., Elder G.J., Strippoli G.F. (2011). Serum levels of phosphorus, parathyroid hormone, and calcium and risks of death and cardiovascular disease in individuals with chronic kidney disease: A systematic review and meta-analysis. JAMA.

[4] Yamada S., Tsuruya K., Taniguchi M., Tokumoto M., Fujisaki K., Hirakata H., Fujimi S., Kitazono T. (2016). Association between serum phosphate levels and stroke risk in patients undergoing hemodialysis: The Q-cohort study. Stroke.

[5] Mittalhenkle A., Gillen D.L., Stehman-Breen C.O. (2004). Increased risk of mortality associated with hip fracture in the dialysis population. Am. J. Kidney Dis..

[6] Russo D., Miranda I., Ruocco C., Battaglia Y., Buonanno E., Manzi S., Russo L., Scafarto A., Andreucci V.E. (2007). The progression of coronary artery calcification in predialysis patients on calcium carbonate or sevelamer. Kidney Int..

[7] Kidney Disease: Improving Global Outcomes (KDIGO) CKD-MBD Update Work Group (2017). KDIGO 2017 clinical practice guideline update for the diagnosis, evaluation, prevention, and treatment of Chronic Kidney Disease–Mineral and Bone Disorder (CKD-MBD). Kidney Int. Suppl..

[8] Goh B.L., Mushahar L., Ching C.H. (2018). 1st Malaysian CKD-MBD & Parathyroidectomy Guidelines and Standard Operating Procedures.

[9] Sahathevan S., Khor B.H., Ng H.M., Gafor A.H.A., Mat Daud Z.A., Mafra D., Karupaiah T. (2020). Understanding development of malnutrition in hemodialysis patients: A narrative review. Nutrients.

[10] St-Jules D.E., Woolf K., Pompeii M.L., Kalantar-Zadeh K., Sevick M.A. (2016). Reexamining the Phosphorus–Protein Dilemma: Does Phosphorus Restriction Compromise Protein Status?. J. Ren. Nutr..

[11] Peter W.L.S., Wazny L.D., Weinhandl E., Cardone K.E., Hudson J.Q. (2017). A review of phosphate binders in chronic kidney disease: Incremental progress or just higher costs?. Drugs.

[12] Hand R.K., Steiber A., Burrowes J. (2013). Renal dietitians lack time and resources to follow the NKF KDOQI guidelines for frequency and method of diet assessment: Results of a survey. J. Ren. Nutr..

[13] Ng E.S.Y., Wong P.Y., Kamaruddin A.T.H., Lim C.T.S., Chan Y.M. (2020). Poor sleep quality, depression and social support are determinants of serum phosphate level among hemodialysis patients in Malaysia. Int. J. Environ. Res. Public Health.

[14] Pollock J.B., Jaffery J.B. (2007). Knowledge of phosphorus compared with other nutrients in maintenance dialysis patients. J. Ren. Nutr..

[15] Cupisti A., Ferretti V., D’Alessandro C., Petrone I., Di Giorgio A., Meola M., Panichi V., Conti P., Lippi A., Caprioli R. (2012). Nutritional knowledge in hemodialysis patients and nurses: Focus on phosphorus. J. Ren. Nutr..

[16] Kalantar-Zadeh K., Gutekunst L., Mehrotra R., Kovesdy C.P., Bross R., Shinaberger C.S., Noori N., Hirschberg R., Benner D., Nissenson A.R. (2010). Understanding sources of dietary phosphorus in the treatment of patients with chronic kidney disease. Clin. J. Am. Soc. Nephrol..

[17] Chan M.W., Cheah H.M., Padzil M.B.M. (2019). Multidisciplinary education approach to optimize phosphate control among hemodialysis patients. Int. J. Clin. Pharm..

[18] Wang A.Y., Okpechi I.G., Ye F., Kovesdy C.P., Brunori G., Burrowes J.D., Campbell K., Damster S., Fouque D., Friedman A.N. (2022). Assessing Global Kidney Nutrition Care. Clin. J. Am. Soc. Nephrol..

[19] Khor B.H., Chinna K., Gafor A.H.A., Morad Z., Ahmad G., Bavanandam S., Visvanathan R., Yahya R., Goh B.L., Bee B.C. (2018). The state of nutrition care in outpatient hemodialysis settings in Malaysia: A nationwide survey. BMC Health Serv. Res..

[20] Karupaiah T., Morad Z. (2007). Perspectives on the nutritional management of renal disease in Asia: People, practice, and programs. J. Ren. Nutr..

[21] Ismail H., Manaf M.R.A., Gafor A.H.A., Zaher Z.M.M., Ibrahim A.I.N. (2019). Economic burden of ESRD to the Malaysian health care system. Kidney Int. Rep..

[22] Kalantar-Zadeh K. (2013). Patient education for phosphorus management in chronic kidney disease. Patient Prefer. Adherence.

[23] Karupaiah T., Swee C.S., Abdullah R. (2001). Developing a nutrition education package for Malaysian hemodialysis patients. J. Ren. Nutr..

[24] Chee W.S.S., Karupaiah T. (2009). Smart Eating for Chronic Kidney Disease-Slowing down Kidney Failure with Protein Counting.

[25] Karupaiah T., Daud Z.A.M., Khor B.H., Sahathevan S., Sualeheen A. (2019). Food Phosphate Guide for Chronic Kidney Disease: What Patients Need to Know, Learn, and Practice.

[26] Leung S., McCormick B., Wagner J., Biyani M., Lavoie S., Imtiaz R., Zimmerman D. (2015). Meal phosphate variability does not support fixed dose phosphate binder schedules for patients treated with peritoneal dialysis: A prospective cohort study. BMC Nephrol..

[27] Young E.W., Akiba T., Albert J.M., McCarthy J.T., Kerr P.G., Mendelssohn D.C., Jadoul M. (2004). Magnitude and impact of abnormal mineral metabolism in hemodialysis patients in the Dialysis Outcomes and Practice Patterns Study (DOPPS). Am. J. Kidney Dis..

[28] Karduck J., Chapman-Novakofski K. (2018). Results of the clinician apps survey, how clinicians working with patients with diabetes and obesity use mobile health apps. J. Nutr. Educ. Behav..

[29] Prest M. (2013). Mobile phone applications for kidney patients. J. Ren. Nutr..

[30] Campbell J., Porter J. (2015). Dietary mobile apps and their effect on nutritional indicators in chronic renal disease: A systematic review. Nephrology.

[31] Kosa S.D., Monize J., D’Souza M., Joshi A., Philip K., Reza S., Samra S., Serrago B., Thabane L., Gafni A. (2019). Nutritional mobile applications for CKD patients: Systematic review. Kidney Int. Rep..

[32] Imtiaz R., Atkinson K., Guerinet J., Wilson K., Leidecker J., Zimmerman D. (2017). A pilot study of OkKidney, a phosphate counting application in patients on peritoneal dialysis. Perit. Dial. Int..

[33] Farfan-Ruiz A.C., Czikk D., Leidecker J., Ramsay T., McCormick B., Wilson K., Zimmerman D. (2020). Multidisciplinary Team Versus a ‘Phosphate Counting’ App for Serum Phosphate Control: A Randomized Controlled Trial. Kidney360.

[34] El Khoury C.F., Crutzen R., Schols J.M., Halfens R.J., Karavetian M. (2020). A dietary mobile app for patients undergoing hemodialysis: Prospective pilot study to improve dietary intakes. J. Med. Internet Res..

[35] Chiang Y.C., Chang Y.P., Lin S.C., Lin C., Hsu P.H., Hsu Y.J., Wu T.J. (2021). Effects of individualized dietary phosphate control program with a smartphone application in hemodialysis patients in Taiwan. Biol. Res. Nurse.

[36] Bobek E., Tversky B. (2016). Creating visual explanations improves learning. Cogn. Res. Princ. Implic..

[37] Kuhlmann M.K. (2006). Management of hyperphosphatemia. Hemodial. Int..

[38] Lieffers J.R., Hanning R.M. (2012). Dietary assessment and self-monitoring with nutrition applications for mobile devices. Can. J. Diet. Pract. Res..

[39] Elliott J.O., Ortman C., Almaani S., Lee Y.H., Jordan K. (2015). Understanding the associations between modifying factors, individual health beliefs, and hemodialysis patients’ adherence to a low-phosphorus diet. J. Ren. Nutr..

[40] Kang H., Park H.A. (2016). A mobile app for hypertension management based on clinical practice guidelines: Development and deployment. JMIR mHealth uHealth.

[41] Chronic Kidney Disease (CKD) Evidence-Based Nutrition Practice Guideline. https://www.andeal.org/topic.cfm?cat=3927&highlight=Chronic%20Kidney%20Disease%20Guideline%202010%20&home=1.

[42] National Kidney Foundation (2013). Dialysis Patients’ Bill of Rights and Responsibilities.

[43] Khor B.H., Sualeheen A., Sahathevan S., Chinna K., Gafor A.H.A., Bavanandan S., Goh B.L., Morad Z., Daud Z.A.M., Khosla P. (2020). Association of dietary patterns with serum phosphorus in maintenance haemodialysis patients: A cross-sectional study. Sci. Rep..

[44] Sualeheen A., Khor B.H., Balasubramanian G.V., Sahathevan S., Ali M.S.M., Narayanan S.S., Chinna K., Daud Z.A.M., Khosla P., Gafor A.H.A. (2020). Habitual dietary patterns of patients on hemodialysis indicate nutritional risk. J. Ren. Nutr..

[45] Cupisti A., Gallieni M., Rizzo M.A., Caria S., Meola M., Bolasco P. (2013). Phosphate control in dialysis. Int. J. Nephrol. Renov. Dis..

[46] Daugirdas J.T., Finn W.F., Emmett M., Chertow G.M., Frequent Hemodialysis Network Trial Group (2011). The phosphate binder equivalent dose. Semin. Dial..

[47] Hruska K.A., Mathew S., Lund R., Qiu P., Pratt R. (2008). Hyperphosphatemia of chronic kidney disease. Kidney Int..

[48] Etikan I., Musa S.A., Alkassim R.S. (2016). Comparison of convenience sampling and purposive sampling. Am. J. Theor. Appl. Stat..

[49] Bertini E., Gabrielli S., Kimani S., Catarci T., Santucci G. Appropriating and assessing heuristics for mobile computing. Proceedings of the Working Conference on Advanced Visual Interfaces.

[50] Zhou L., Bao J., Setiawan I.M.A., Saptono A., Parmanto B. (2019). The mHealth APP usability questionnaire (MAUQ): Development and validation study. JMIR mHealth uHealth.

[51] Chen S.C., Liu M.L., Lin C.P. (2013). Integrating technology readiness into the expectation–confirmation model: An empirical study of mobile services. Cyberpsychol. Behav. Soc. Netw..

[52] Baharum A., Jaafar A. User interface design: A study of expectation-confirmation theory. Proceedings of the 5th International Conference on Computing and Informatic.

[53] Lim J.-H., Chinna K., Khosla P., Karupaiah T., Daud Z.A.M. (2020). Understanding How Nutrition Literacy Links to Dietary Adherence in Patients Undergoing Maintenance Hemodialysis: A Theoretical Exploration Using Partial Least Squares Structural Equation Modeling. Int. J. Environ. Res. Public Health.

[54] Lambert K., Mullan J., Mansfield K., Owen P. (2017). Should we recommend renal diet–related apps to our patients? An evaluation of the quality and health literacy demand of renal diet–related mobile applications. J. Ren. Nutr..

[55] Pinto L.C.S., Andrade M.C., Chaves R.O., Lopes L.L.B., Maués K.G., Monteiro A.M., Nascimento M.B., Barros C.A.V. (2020). Development and validation of an application for follow-up of patients undergoing dialysis: NefroPortátil. J. Ren. Nutr..

[56] Elder G.J., Malik A., Lambert K. (2018). Role of dietary phosphate restriction in chronic kidney disease. Nephrology.

[57] Welch J., Dowell S., Johnson C.S. (2007). Feasibility of using a personal digital assistant to self-monitor diet and fluid intake: A pilot study. Nephrol. Nurs. J..

[58] Koprucki M., Piraino B., Bender F., Snetselaar L., Hall B., Stark S., Sevick M.A. (2010). RCT of Personal Digital Assistant (PDA) supported dietary intervention to reduce sodium intake in PD. Am. J. Kidney Dis..

[59] Stark S., Snetselaar L., Piraino B., Stone R.A., Kim S., Hall B., Burke L.E., Sevick M.A. (2011). Personal digital assistant-based self-monitoring adherence rates in 2 dialysis dietary intervention pilot studies: BalanceWise-HD and BalanceWise-PD. J. Ren. Nutr..

[60] Cueto-Manzano A.M., Gallardo-Rincón H., Martínez-Ramírez H.R., Cortés-Sanabria L., Rojas-Campos E., Tapia-Conyer R., Martínez P., Cerrillos I., Andrade J., Medina M. (2015). A pilot study of a mobile phone application to improve lifestyle and adherence of patients with kidney disease. J. Telemed. Telecare.

[61] Connelly K., Siek K.A., Chaudry B., Jones J., Astroth K., Welch J.L. (2012). An offline mobile nutrition monitoring intervention for varying-literacy patients receiving hemodialysis: A pilot study examining usage and usability. J. Am. Med. Inform. Assoc..

[62] Welch J.L., Astroth K.S., Perkins S.M., Johnson C.S., Connelly K., Siek K.A., Jones J., Scott L.L. (2013). Using a mobile application to self-monitor diet and fluid intake among adults receiving hemodialysis. Res. Nurs. Health.

[63] Lee J.Y., Wong C.P., Lee S.W.H. (2020). m-Health views and perception among Malaysian: Findings from a survey among individuals living in Selangor. Mhealth.

[64] DiMatteo M.R., Haskard K.B., Williams S.L. (2007). Health beliefs, disease severity, and patient adherence: A meta-analysis. Med. Care.

[65] Weinstein N.D., Sandman P.M., Blalock S.J., Sweeny K., Robbins M.L., Cohen L.M. (2020). The precaution adoption process model. The Wiley Encyclopedia of Health Psychology.

