# Supplementary material for: A Mobile App for Triangulating Strategies in Phosphate Education Targeting Patients with Chronic Kidney Disease in Malaysia: Development, Validation, and Patient Acceptance
Source: Healthcare (Basel). 2022 Mar 14;10(3):535. doi: 10.3390/healthcare10030535 (PMC8950478; doi:10.3390/healthcare10030535)
Supplement: Supplementary file 1 [file healthcare-10-00535-s001.zip › healthcare-1609576-supplementary.pdf]

Supplementary Tables & Figures

Table S1. Strategies of hyperphosphatemia management for PMA development

Health Beliefs	Strategies	Domains
Perceived Susceptibility	Educate on disease/condition risk factors	Phosphate & Hyperphosphatemia<br>Dialysis<br>Phosphate binder<br>Dietary phosphate<br>Lifestyle on dialysis<br>Responsibility as a dialysis patient
Perceived Severity	Educate on the consequences of the disease/condition<br>Educate on the potential impact on the patient's lifestyle	
Perceived Benefits	Educate on the benefits of nutrition therapy and physical activity<br>Imagine the quality of life	
Perceived Barriers	Educate on barriers of nutrition therapy and physical activity	
Cues to Action	Provide reminder<br>Emphasis on how-to-education<br>Link current symptoms to disease/condition	
Self-Efficacy	Introduce alternatives and choices<br>Goal setting<br>Self-monitoring	

Table S2. Criteria of management domains for hyperphosphatemia

Domains	Criteria
Phosphate and Hyperphosphatemia	Source and function of phosphate.<br>Factors and health risks of hyperphosphatemia.<br>Target serum phosphorus level is 1.18-1.78 mmol/L.<br>Management for hyperphosphatemia focusing on adequate dialysis, phosphate binder intake and practice of reducing dietary phosphate intake (overview).
Dialysis	Function and type of dialysis.<br>Amount of phosphate removed via dialysis.<br>Dialysis adequacy indicator (Kt/V).<br>Health complications of inadequate dialysis.
Phosphate binder	Type of phosphate binders.<br>Mechanism of phosphate binder.<br>How and when to take the phosphate binder.<br>Contraindication for the phosphate binder.<br>Risks of hypercalcemia from calcium-based phosphate binder.<br>Reminder to take phosphate binder.<br>Phosphate binder need according to dietary phosphate intake.
Dietary Phosphate	Source and amount of dietary phosphate.<br>Source of inorganic phosphate and reading food label.<br>Source of animal and plant-based phosphate (organic)<br>Absorption level of phosphate.<br>Type of food with acceptable phosphate-to-protein ratio.<br>Risks of hyperkalemia from plant-based food.<br>Tip to reduce dietary phosphate during food preparation.
Lifestyle on dialysis	Eat balanced meals with a variety of food, with consideration of specific nutrient needs.<br>Limit or avoid alcohol consumption.

	Stay away from cigarette smoke.<br>Encourage to move more outside of dialysis session.<br>Monitor sugar and blood pressure level at home.<br>Be careful with supplements, herbal and traditional products.<br>Take care of personal hygiene.
Responsibility as a dialysis patient	Ask health care providers whenever in doubt.<br>Take part in treatment decision-making and cooperate fully.<br>Arrange transportation to the treatment centre.<br>Be on time.<br>Make necessary arrangements for dialysis in advance when travelling long distances.<br>Apply for funding to cover the medical expenses of treatment.<br>Recognise medication and take medication as prescribed.<br>Follow the prescribed diet and the amount of fluid allocated.<br>Resume the normal activities<br>Understand your own needs and responsibilities.

Table S3. Characteristics of Expert Reviewers

Characteristics	n (%)
Professional background	
Physician	5 (38.5)
Pharmacist	2 (15.4)
Dietitian	6 (46.2)
Affiliation by sector	
Ministry of Health	7 (53.8)
Ministry of Education	4 (30.8)
Private	2 (15.4)
Practice (Year)	
≥20	5 (38.5)
11-19	7 (53.8)
8-10	1 (7.7)
Age Group	
30-39	4 (30.8)
40-49	4 (30.8)
50-59	5 (38.5)
Ethnicity	
Chinese	6 (46.2)
Malay	4 (30.8)
Indian	3 (23.1)
Gender	
Female	9 (69.2)
Male	4 (30.8)

Table S4. Knowledge-based accuracy feedback from the expert panel

No.	Domain	Statement			
		Total	Corrected	Improved	Enhanced
1	Phosphate & hyperphosphatemia	6	2	2	2
2	Dialysis	2	-	-	2
3	Phosphate binder	10	2	3	5
4	Dietary	11	2	6	3

5	Lifestyle on dialysis	12	1	7	4
6	Responsibility as a dialysis patient	2	-	2	-
	Total	43	7	20	16

Table S5. Knowledge-based accuracy feedback from the expert panel

No.	Components	Severity Ranking Scale[a]					
		**Total**	**0**	**1**	**2**	**3**	**4**
1	Visibility of system status	3	-	1	-	2	-
2	Match between system and the real world	8	-	1	6	1	-
3	Consistency and mapping	5	-	-	2	3	-
4	Good ergonomics and minimalist design	2	-	-	1	1	-
5	Ease of input, screen readability and glanceability	14	-	3	3	8	-
6	Flexibility, efficiency of use and personalisation	5	-	2	3	-	-
7	Aesthetic, privacy and social conventions	7	-	5	1	1	-
8	Realistic error management	2	-	0	1	1	-
	TOTAL	46	-	12	17	17	-

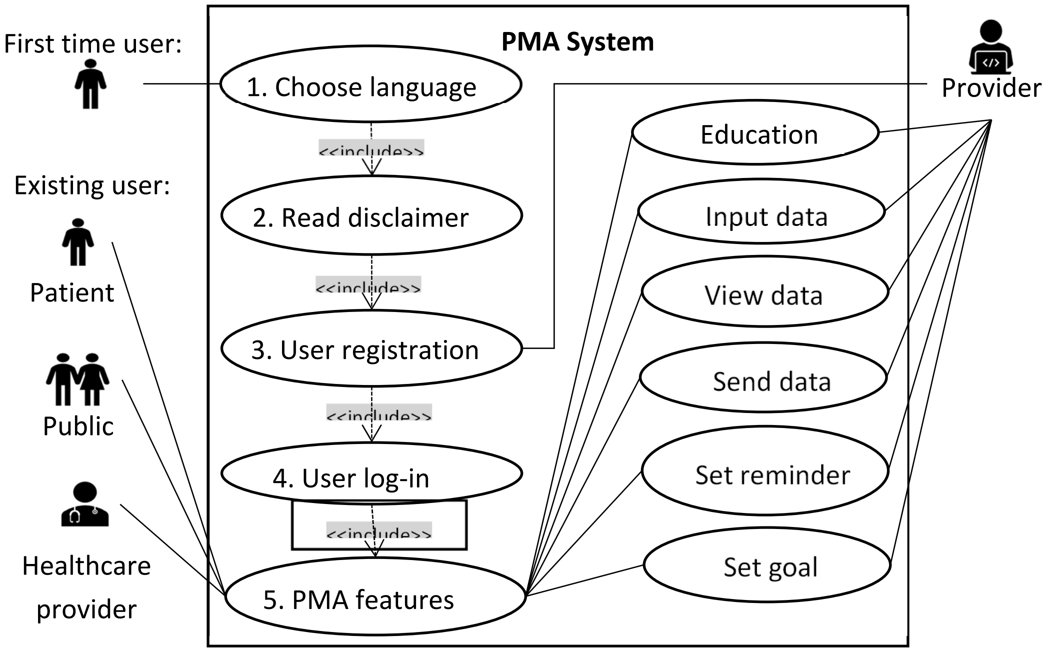

Figure S1. Use-case diagram of the PMA

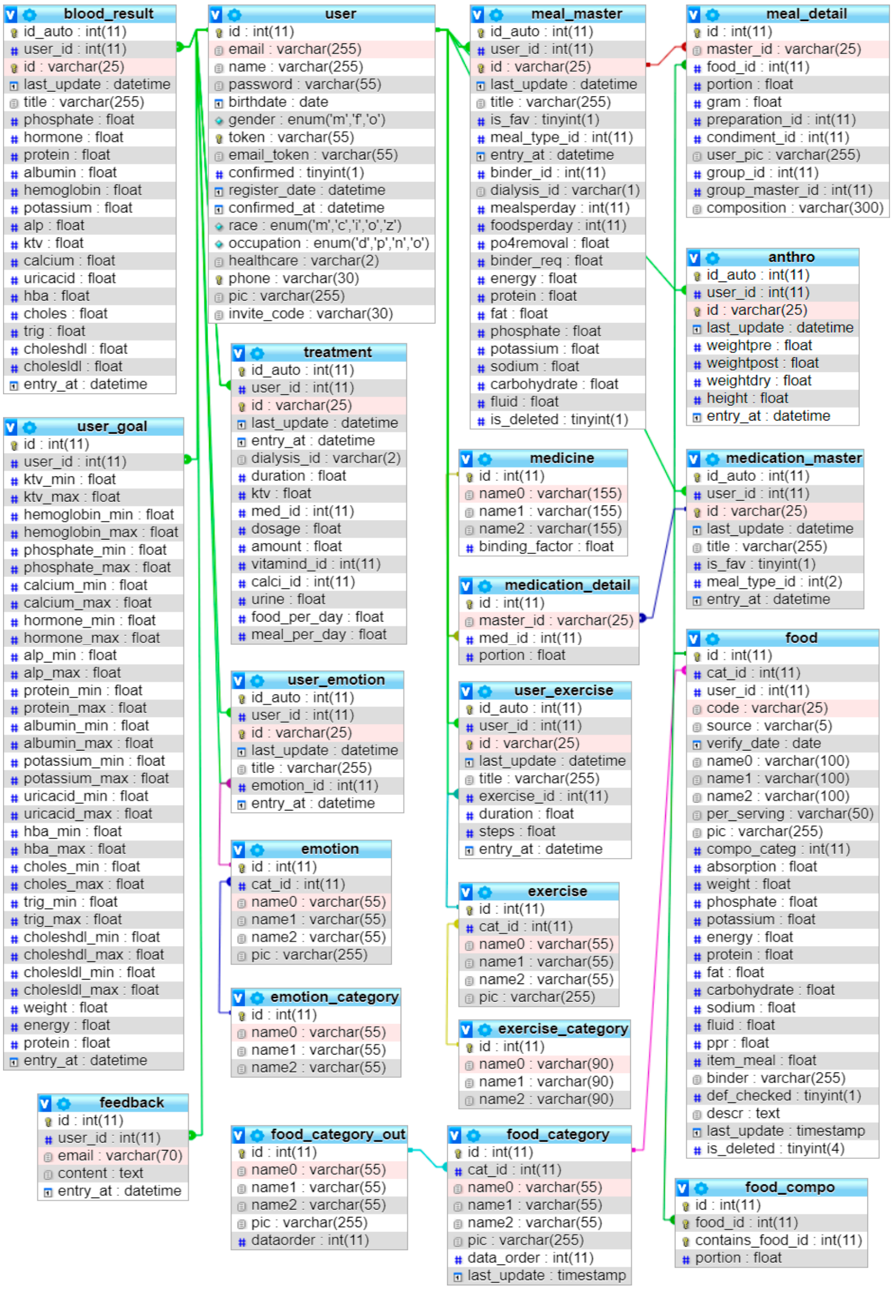

Figure S2. Entity-relation diagram of the PMA
